# Siglec‐7 and Siglec‐9 expression in primary triple negative and oestrogen receptor positive breast cancer and *in vitro* signalling

**DOI:** 10.1002/cti2.1524

**Published:** 2024-09-06

**Authors:** Eline JH van Houtum, Anne HC Valk, Daniel Granado, Jasper Lok, Lune van den Bogaard, Naomi Remkes, Jesper van Eck van der Sluijs, Paul N Span, Lenneke AM Cornelissen, Gosse J Adema

**Affiliations:** ^1^ Radiotherapy & OncoImmunology Laboratory, Department of Radiation Oncology Radboud University Medical Center Nijmegen The Netherlands

**Keywords:** immune checkpoint receptors, immunotherapy, oestrogen receptor positive breast cancer, Siglecs, triple negative breast cancer

## Abstract

**Objectives:**

PD‐1/PD‐L1 immune checkpoint blockade can be an effective treatment for advanced breast cancer patients. However, patients with oestrogen receptor positive (ER+) tumors often display only low lymphocyte infiltration, while a large part of triple negative (TN) breast tumors does not generate an effective immunotherapy response. Therefore, new treatment strategies have to be developed. Here, we investigate Siglec‐7 and Siglec‐9 as novel ITIM‐bearing inhibitory immune checkpoint receptors similar to PD‐1, but expressed on a broader range of immune cells.

**Methods:**

We assessed Siglec‐7 and Siglec‐9 (ligand) expression in TN and ER+ breast cancer tumors and their breast cancer cell line‐induced signalling.

**Results:**

We report that Siglec‐7 and Siglec‐9 are highly expressed in TN tumors, and to a low extent in ER+ tumors. Siglec‐7 was observed on myeloid cells, T cells, and NK cells and Siglec‐9 preferentially on myeloid cells. Expression of sialoglycans, including Siglec‐7 and Siglec‐9 ligands, was observed in both TN and ER+ breast cancer tissue sections. Expression levels of Siglec‐7 and Siglec‐9 ligands were higher on *in vitro* cultured TN cell lines than ER+ cell lines. Importantly, by applying chimeric Siglec‐7 reporter cells, we showed the induction of Siglec‐7 signalling by multiple TN cell lines, but only by one ER+ cell line. Moreover, Siglec‐7 signalling is directly related to Siglec‐7 ligand expression levels of breast cancer cell lines.

**Conclusion:**

These data imply that immunotherapy targeting Siglec receptors may be particularly interesting for TN breast cancer patients not responding to current treatment strategies with tumors displaying high immune cell infiltration.

## Graphical abstract

In this study, we found high Siglec‐7 and Siglec‐9 (ligand) expression in triple negative breast cancer, and to a lower extent in oestrogen receptor positive breast cancer. Moreover, signalling experiments demonstrated that Siglec‐7 signalling is induced by triple negative breast cancer cell lines. Immunotherapy targeting these Siglec receptors may, therefore, be particularly interesting for triple negative breast cancer patients who are not responding to current treatment strategies.

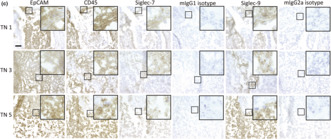

## Introduction

Tumor cells have adapted various ways to create an immunosuppressive tumor microenvironment (TME), such as secretion of suppressive molecules and expression of cell surface proteins that interact with immune checkpoint receptors on immune cells to cause immune cell inhibition.^1^ Immune checkpoint therapy is an anti‐tumor treatment that prevents interaction of an immune checkpoint receptor on immune cells with its ligand, and this treatment has shown effective results in the clinic.^2^, ^3^ A widely used checkpoint therapy is blocking the PD‐1/PD‐L1 interaction, to prevent PD‐1‐mediated T cell inhibition.^2^, ^4^, ^5^ However, many tumors do not respond to such T cell targeting checkpoint therapies and, therefore, development of alternative strategies is required.^3^, ^4^, ^6^

Triple negative (TN) breast cancer is typically known for its aggressive behaviour, characterised by a high risk for metastasis, high disease recurrence and low survival rates.^7^, ^8^ Molecularly, it is defined by the absence of expression of the oestrogen receptor (ER), progesterone receptor and HER2.^9^ Other features that are linked to this breast cancer subtype are its higher mutational load, high infiltration by tumor‐infiltrating lymphocytes (TILs) and high expression of PD‐L1 compared to other breast cancer subtypes.^10^, ^11^, ^12^ TN breast cancer contributes to 15–20% of the total breast cancer cases and is associated with the poorest prognosis of all subtypes.^8^, ^9^ Recently, the development of immunotherapy has significantly improved treatment outcomes of TN breast cancer.^13^ Several studies have shown objective response rates ranging from 5% to 30%.^14^ Especially the combination of high TIL infiltration, high mutational load, and high PD‐L1 expression makes TN breast cancer a promising candidate for immunotherapy. However, 20% of TN breast cancer patients does not express significant levels of PD‐L1, which makes PD‐1/PD‐L1 blockade ineffective.^12^

Compared to TN breast cancer, the ER positive (ER+) breast cancer subtype accounts for a larger proportion of breast cancer cases, and has a higher 5‐year overall survival with current therapies, mainly hormonal therapy or chemotherapy.^15^, ^16^ For those patients who do not respond to current therapy strategies, PD‐1/PD‐L1 targeted immunotherapy does not offer an alternative strategy as most ER+ tumors have low TIL infiltration.^17^

New therapies have to be established for patients with PD‐L1 negative TN breast cancer or ER+ tumors with limited TIL infiltration for whom current treatment options are ineffective. Current immunotherapy strategies mainly target checkpoint receptors associated with T cell activation.^18^ However, activation of other immune cells in the TME, such as myeloid cells or natural killer (NK) cells, is also modulated by ligation of checkpoint receptors.^19^ Moreover, co‐expression of other inhibitory checkpoint receptors on PD‐1 expressing T cells might inhibit T cell activation despite application of PD‐1 targeted checkpoint blockade. Targeting such checkpoint receptors could be an alternative or complimentary approach for treatment of cancer. A family of checkpoint receptors that is expressed by a wide variety of immune cells are the Sialic acid‐binding immunoglobulin‐like lectins (Siglecs).^20^, ^21^, ^22^ These fourteen different cell surface receptors are expressed by immune cells from both the lymphoid as well as the myeloid lineages. All Siglec receptors carry an extracellular sialic acid‐binding domain, through which they bind sialoglycans, which are glycans that carry at least one sialic acid residue. Intracellularly, most Siglec receptors harbour an immunoreceptor tyrosine‐based inhibitory motif (ITIM) and an ITIM‐like domain, similar to PD‐1, which cause immune cell inhibition upon ligand binding.^20^ Therefore, these inhibitory Siglecs resemble the PD‐1 checkpoint receptor both in structure as well as function.^22^, ^23^, ^24^ Tumor cells are known to overexpress sialoglycan ligands causing Siglec‐dependent immune cell inhibition.^25^

Two interesting inhibitory Siglec receptors for immunotherapy are Siglec‐7 and Siglec‐9. They are highly homologous, and expression has been reported on an overlapping set of immune cell subsets, such as NK cells, monocytes and macrophages.^26^, ^27^, ^28^ Therefore, targeting these receptors could be a promising strategy to potentiate the immune system on both myeloid and lymphoid cells. The inhibitory roles of Siglec‐7 andSiglec‐9 have been clearly demonstrated in the literature.^29^, ^30^ More importantly, recent research has shown that these Siglec receptors can be highly expressed by immune cells infiltrating in cancer, including breast cancer.^31^ However, the presence of Siglec ligands does not necessarily lead to Siglec signalling.^32^ Besides the need for additional information on the Siglec and sialoglycan expression patterns in breast cancer, studies on the occurrence of Siglec signalling upon breast tumor cell encounter are currently lacking. Here we report on the expression of Siglec‐7 and Siglec‐9 and their ligands in TN and ER+ tumors and on Siglec‐7 and Siglec‐9 signalling induced by breast cancer cells to determine their potential as targets for future immunotherapy in breast cancer.

## Results

### Higher Siglec‐7 and Siglec‐9 expression in TN than in ER+ tumors

**Figure 1:**
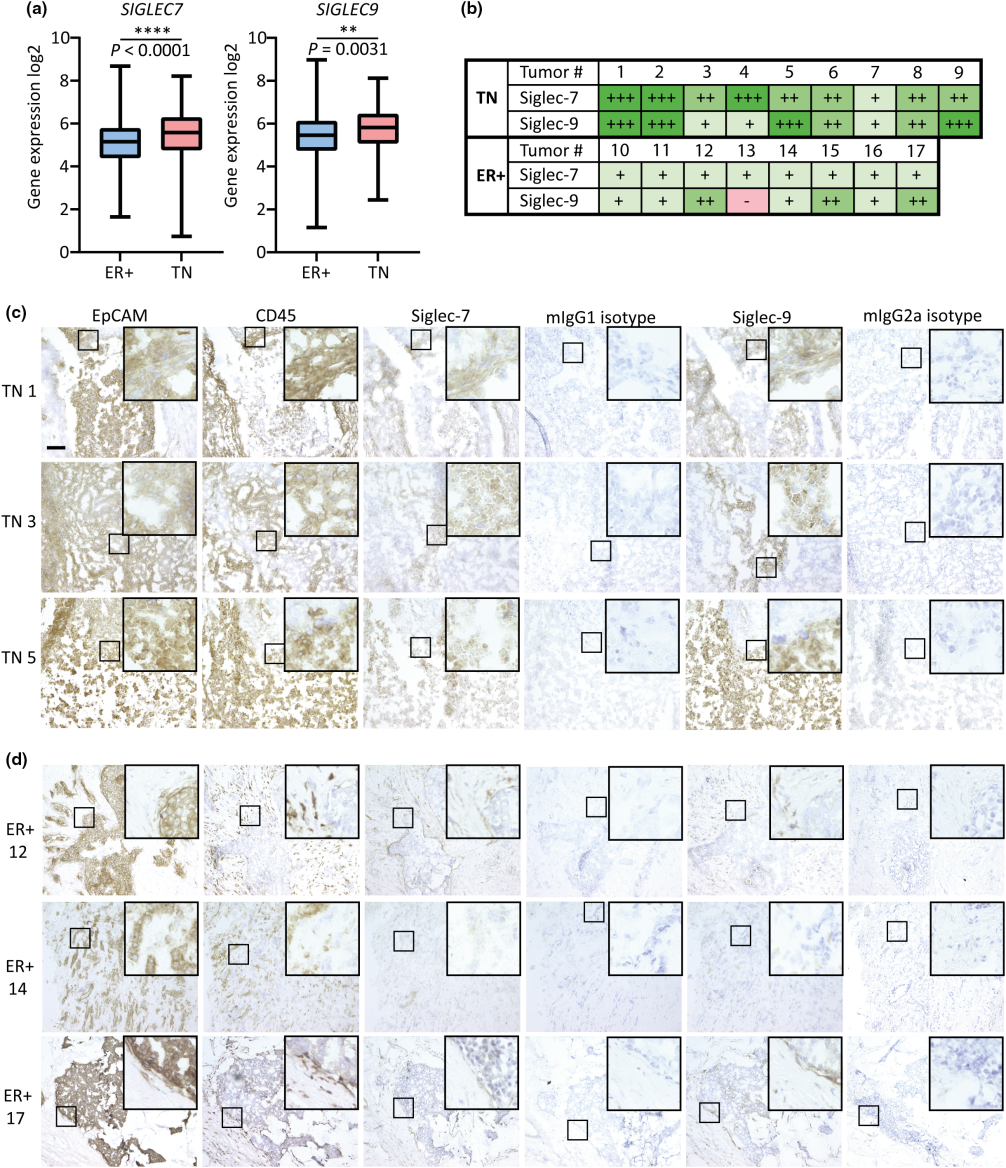
Siglec‐7 and Siglec‐9 expression is higher in TN tumors than ER+ tumors. **(a)** Expression of *SIGLEC7* and *SIGLEC9* from TN (*n* = 141) and ER+ (*n* = 808) breast cancer samples was retrieved from the TCGA breast cancer cohort using the UCSC Xena platform. A Mann–Whitney *U*‐test was performed to assess significance (*P* < 0.01**,  *P* < 0.0001****). **(b)** Extent of Siglec‐7 and Siglec‐9 expression in 9 TN and 8 ER+ breast tumors was scored by two researchers. ‘−’ indicates negative, ‘+’ slightly positive, ‘++’ positive and ‘+++’ highly positive. **(c)** TN tumors and **(d)** ER+ tumors were stained for EpCAM, CD45, Siglec‐7, Siglec‐9, mIgG1 (isotype control for EpCAM, CD45 and Siglec‐7) and mIgG2a (isotype control for Siglec‐9). Stainings of 3 representative TN tumors and ER+ tumors are shown. The scale bar represents 100 μm.

To examine expression levels of *SIGLEC7* and *SIGLEC9* in ER+ and TN breast cancer, RNA expression in the TCGA breast cancer cohort was analysed (Figure 1a). Both *SIGLEC7* and *SIGLEC9* were expressed in significantly higher levels in TN than in ER+ breast cancer samples. Next, Siglec‐7 and Siglec‐9 protein expression was assessed. Immunohistochemical stainings for Siglec‐7 and Siglec‐9 of nine TN breast tumors and eight ER+ breast tumors were performed (Figure 1b–d and Supplementary figure 1). In parallel, stainings for EpCAM and the pan immune cell marker CD45 were used to identify tumor cells and to assess immune cell infiltration, respectively. TN tumors showed a higher overall Siglec‐7 and Siglec‐9 protein expression than ER+ tumors (Figure 1b–d). Isotype controls were negative for all antibodies used. Importantly, EpCAM and CD45 stainings of consecutive tumor sections verified a much higher immune cell infiltration in TN (Figure 1c) tumors than ER+ tumors (Figure 1d).

### Siglec‐7 is expressed on CD3^+^ cells, CD11b^+^ cells, and on CD56^+^ cells and Siglec‐9 on CD11b^+^ cells

**Figure 2:**
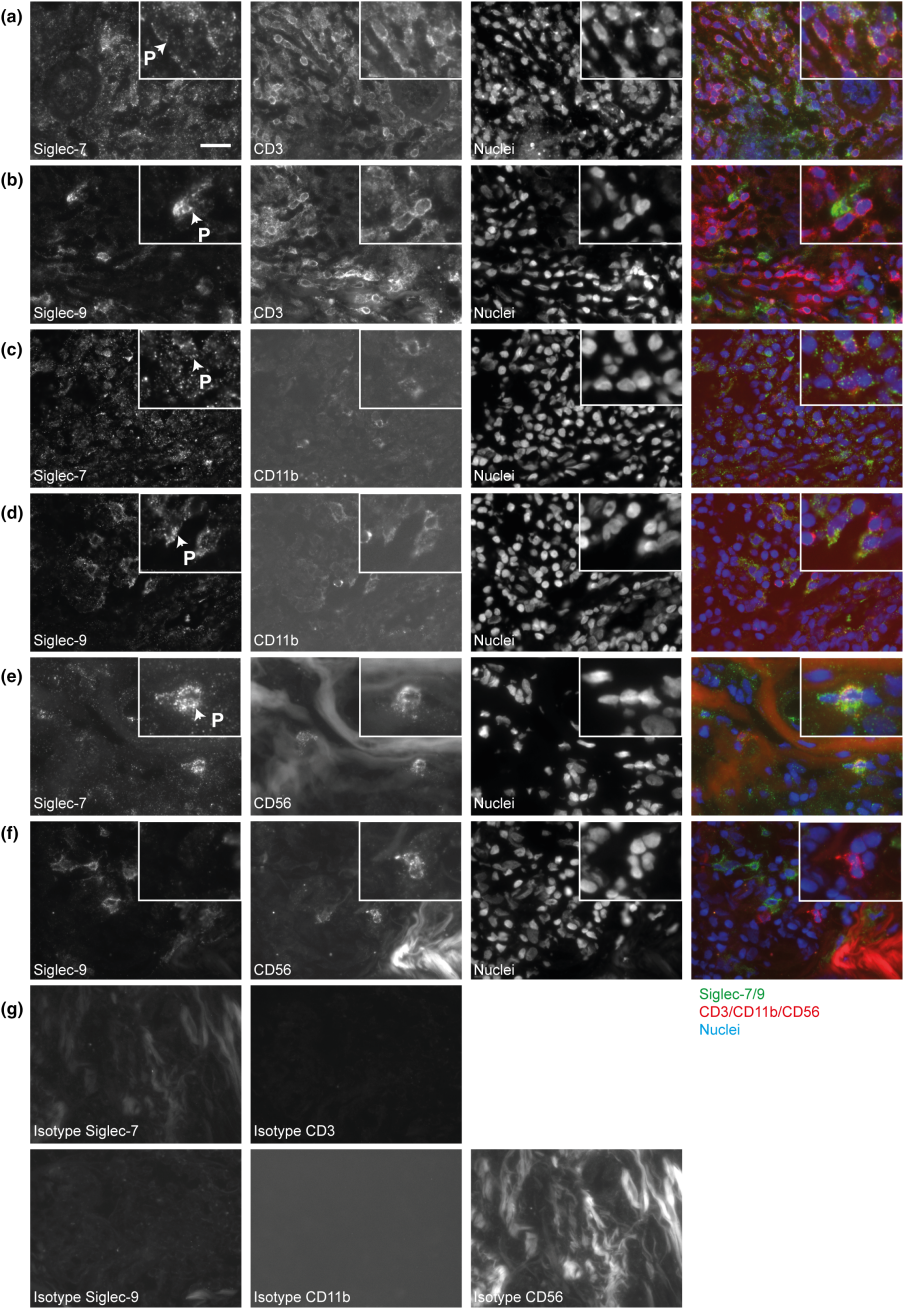
Siglec‐7 is expressed on CD3^+^ cells, CD56^+^ cells and CD11b^+^ cells, and Siglec‐9 is expressed on CD11b^+^ cells. Double fluorescent stainings for Siglec‐7 or Siglec‐9 with either CD3, CD11b, or CD56 and respective isotype controls were performed on breast cancer sections. Panels indicate the following combined markers on one tumor section: **(a)** Siglec‐7 and CD3, **(b)** Siglec‐9 and CD3, **(c)** Siglec‐7 and CD11b, **(d)** Siglec‐9 and CD11b, **(e)** Siglec‐7 and CD56, **(f)** Siglec‐9 and CD56 and **(g)** isotype controls. Data from a representative TN tumor are shown. The ‘P’ and arrow indicate punctuated Siglec‐7 or ‐9. **(a–g)** Represent different regions within the tumor section, so only the four panels within one subfigure can be compared. The scale bar indicates 20 μm.

Next, adjacent tumor sections were stained for EpCAM and the immune cell markers (CD45, CD3, CD66b, CD56 and CD11b), to determine the immune cells that infiltrate the breast tumors. Both TN and ER+ tumors were infiltrated by CD3^+^ (T cells), CD11b^+^ (myeloid cells) and CD56^+^ (NK cells) immune cells, although ER+ tumors to a lesser extent than TN tumors (Supplementary figure 2a, b). CD66b (granulocytes) was not detected in most tumors. Immunofluorescent double stainings of Siglec‐7 or Siglec‐9 with either CD3, CD11b or CD56 were performed (Figure 2a–g), and revealed that CD3^+^, CD56^+^ and CD11b^+^ cells display expression of Siglec‐7 (Figure 2a, c and e). Interestingly, the majority (> 75%) of CD3^+^ T cells displayed Siglec‐7 expression, almost all (> 90%) of the CD56^+^ cells and most (75%) of the CD11b^+^ cells showed Siglec‐7 expression. Siglec‐9 was expressed on almost all (> 90%) of the CD11b^+^ cells but was essentially absent on CD3^+^ or CD56^+^ cells (Figure 2d). Interestingly, heterogeneous Siglec‐7 and Siglec‐9 staining patterns were observed, including a more punctuated appearance on the cell membrane.

### Siglec‐7 and Siglec‐9 ligands are present in both TN and ER+ tumors

To examine the presence of acidic glycoproteins, such as sialomucins, breast tumor sections were stained with alcian blue (Supplementary figure 3a, b). Alcian blue staining was clearly present in TN tumors (Supplementary figure 3a) as well as in ER+ tumors (Supplementary figure 3b). Besides, the presence of sialic acids was further verified in both tumor types, using the MALII lectin (a protein that binds α‐2,3 linked sialic acid) and the SNAI lectin (a protein that binds α‐2,6 linked sialic acid) (Supplementary figure 4a, b).

**Figure 3:**
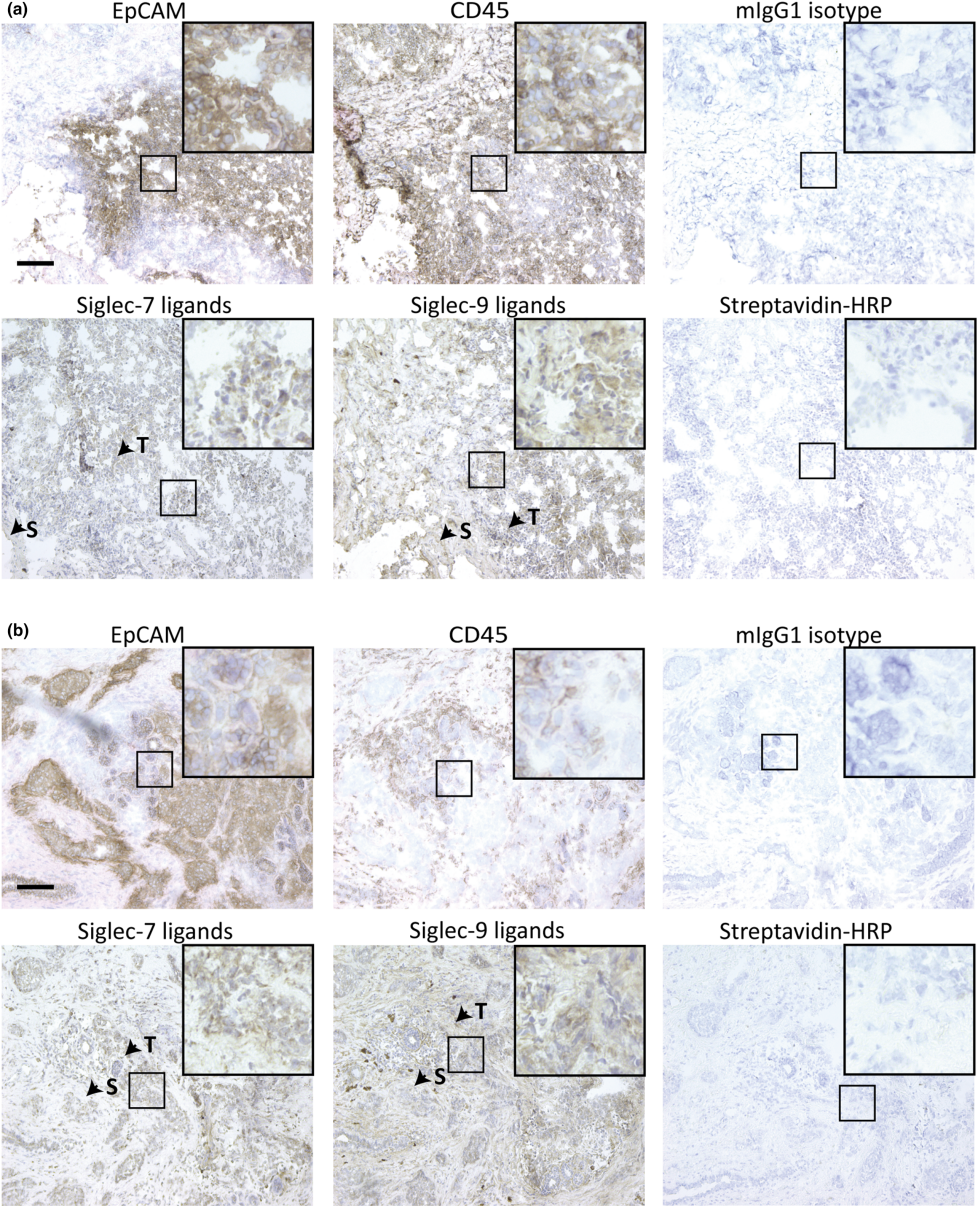
TN and ER+ tumors express high levels of Siglec‐7 ligands and Siglec‐9 ligands. Siglec‐7 and Siglec‐9 ligands were stained using biotinylated Siglec‐7 or Siglec‐9 in **(a)** TN tumors and **(b)** ER+ tumors. Sections were additionally stained for EpCAM, CD45 and their isotype control mIgG1. For each subtype, a representative tumor is shown. As control for Siglec ligands, the sections were stained with streptavidin‐HRP. ‘T’ indicates tumor cells and ‘S’ indicates stroma. The scale bar equals 20 μm.

Importantly, after investigating general sialoglycoprotein expression, the expression of specific Siglec‐7 and Siglec‐9 ligands in TN tumors and ER+ tumors was determined. TN (Figure 3a) and ER+ (Figure 3b) tumors were stained using biotinylated human Siglec‐7 or Siglec‐9 fusion protein, and these tumors were also assessed for EpCAM and CD45 expression. Interestingly, both subtypes displayed high expression of Siglec‐7 and Siglec‐9 ligands. Siglec‐7 ligands were predominantly found on the EpCAM^+^ tumor cells and to a lesser extent on tumor stroma, whereas Siglec‐9 ligands were expressed on both the EpCAM^+^ tumor cells and also highly on the tumor stroma.

### TN and ER+ tumor cell lines expressing high Siglec‐7 ligands can induce Siglec‐7 signalling

**Figure 4:**
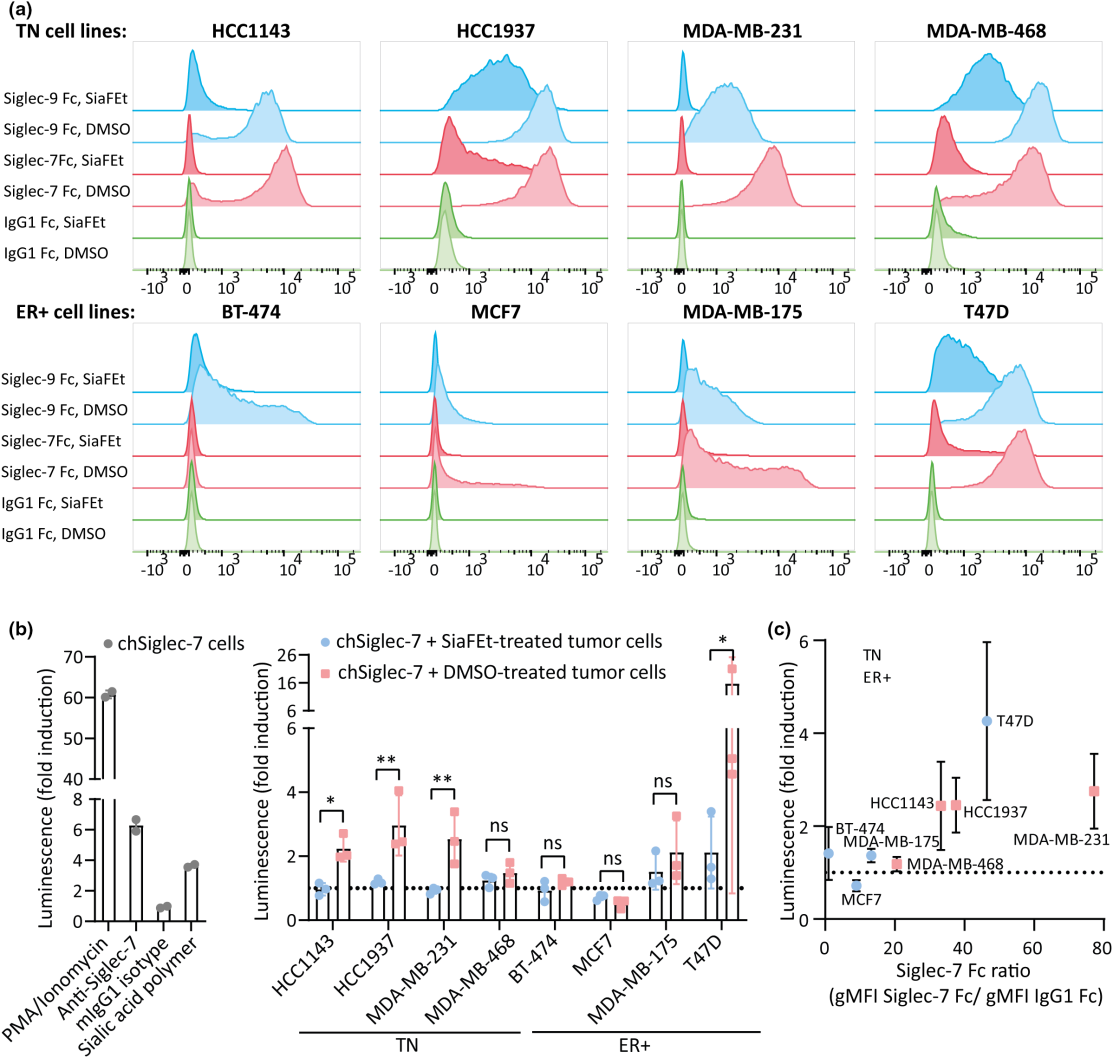
chSiglec‐7 signalling is induced by multiple TN cell lines and one ER+ tumor cell line. **(a)** Siglec‐7 Fc and Siglec‐9 Fc ligands on SiaFEt‐ or DMSO‐treated breast cancer cell lines were determined using flow cytometry, using IgG1 Fc as isotype control. Cell lines were treated for 3 days with 31.5 μM SiaFEt or DMSO as vehicle control. Representative data of *n* = 2 or 3 experiments are shown. **(b)** chSiglec‐7 cells were validated for functionality by culture with PMA/ionomycin, by culture on wells coated with anti‐Siglec‐7 antibody or its respective mIgG1 isotype control or by culture with sialic acid polymer (left panel). 2 Technical replicates per condition are shown of a representative luciferase assay. chSiglec‐7 Jurkat/MA cells were co‐cultured with SiaFEt‐ or DMSO‐treated tumor cell lines and subsequently chSiglec‐7 signalling was assessed by luciferase assays (right panel). Luciferase assays were repeated 3 times, each containing 2–3 technical replicates per condition. Luminescence values are normalised to those of unstimulated chSiglec‐7. Bars represent mean ± SD of 3 luciferase assays. Matched one‐way ANOVAs were performed on log‐transformed raw data (adjusted *P* < 0.05*, *P* < 0.01**). **(c)** Correlation between Siglec‐7 ligand expression and chSiglec‐7 induction by the cell lines. Luminescence values of chSig7 co‐culture with DMSO‐treated tumor cells are normalised to those of co‐culture with SiaFEt‐treated tumor cells. Dots represent mean ± SD of 3 luciferase assays.

Still little is known regarding actual signalling of Siglecs upon encounter of sialoglycan ligands on tumor cells. Therefore, we studied the expression and signalling inducing capacity of Siglec‐7 and Siglec‐9 ligands as present on a set of TN and ER+ tumor cell lines. Siglec‐7 and Siglec‐9 ligands were readily detected on both the TN tumor cell lines (HCC1143, HCC1937, MDA‐MB‐231, MDA‐MB‐468) and the ER+ tumor cell lines (BT‐474, MCF7, MDA‐MB‐175, T47D) (Figure 4a and Supplementary figure 5). The Siglec‐7 and Siglec‐9 ligand expression levels, however, were higher on the TN cell lines than on the ER+ cell lines, with the exception of the ER+ cell line T47D.

The ability of the different tumor cell lines to induce Siglec‐7 signalling was assessed, using our recently established chimeric Siglec‐7 (chSiglec‐7) Jurkat/MA signalling tool.^32^ These Jurkat/MA NFAT‐luciferase reporter cells harbour a chSiglec‐7 receptor that contains the extracellular domains of the Siglec‐7 coupled to the transmembrane and intracellular domains of CD3ζ. Upon Siglec‐7 triggering by sialic acid ligand binding, the intracellular signalling pathway is initiated, resulting in transcription of luciferase. Functionality of the luciferase reporter system was verified by stimulation with PMA and ionomycin and Siglec‐7/sialic acid dependent signalling was shown by culture with coated anti‐Siglec‐7 antibody and by culture with a Neu5Ac sialic acid polymer as we have previously reported on (Figure 4b).^32^ The chSiglec‐7 Jurkat/MA reporter cells were co‐cultured with the TN and ER+ breast tumor cells treated with DMSO as vehicle control. The same tumor cells lacking sialic acid ligands following treatment with the sialyltransferase inhibitor SiaFEt were used as controls. Multiple TN tumor cell lines (HCC1143, HCC1937 and MDA‐MB‐231) were found to significantly initiate chSiglec‐7 signalling, whereas the sialic acid high T74D cell line was the only ER+ cell line able to do so (Figure 4b and Supplementary figure 6). The MDA‐MB‐468 TN cell line displaying lower Siglec‐7 ligand levels was the only TN cell line unable to induce chSiglec‐7 signalling. Strikingly, plotting Siglec‐7 ligand expression levels against luminescence values revealed that beyond a certain threshold of Siglec‐7 Fc staining, Siglec‐7 ligand expression aligned well with the ability to induce Siglec‐7 signalling (Figure 4c).^32^ As most TN and ER+ cell lines also displayed Siglec‐9 ligands, we tested their capacity to induce signalling of a novel chimeric Siglec‐9 Jurkat/MA reporter cell line. None of the breast cancer cell lines, however, were able to induce signalling of the chimeric Siglec‐9 receptor, while Siglec‐9 antibodies and a Siglec‐9 ligand containing polymer used as a positive controls readily induced chSiglec‐9 signalling in these cells. chSiglec‐9R120A, a chimeric Siglec‐9 receptor that is unable to bind sialic acid, did not signal upon culture with the Siglec‐9 ligand polymer, thereby verifying sialic acid‐dependence of the system (Supplementary figure 7).

## Discussion

Siglecs are potential glyco‐immune checkpoints in cancer. Analysis of Siglec‐7 and Siglec‐9 expression and function in TN and ER positive breast cancer showed that TN tumors express higher Siglec‐7 and Siglec‐9 RNA and protein levels as compared to ER+ tumors. Siglec‐7 expression in the breast cancer TME was found on T cells, myeloid cells and NK cells in human breast tumors, whereas Siglec‐9 was observed on myeloid cells. Siglec‐7 and Siglec‐9 ligands were present in both TN and ER+ tumor subtypes *in situ* as well by a set of TN and ER+ tumor cell lines. Importantly, multiple TN tumor cell lines were able to trigger chSiglec‐7 signalling, but only one ER+ cell line induced Siglec‐7 signalling. The ability to induce chSiglec‐7 signalling aligned with the expression level of Siglec‐7 ligands on the breast cancer cell lines.

In line with previous literature, we have observed more immune cell infiltration in TN tumors compared to ER+ tumors, which makes the first subtype more promising for immunotherapy treatment.^33^ This has been suggested to be influenced by a difference in chemokine‐mediated immune cell recruitment or by the difference in mutational burden and associated higher display of neoantigens.^9^, ^10^, ^13^, ^34^, ^35^ Analysis of the immune cells expressing Siglec‐7 and Siglec‐9 in the breast cancer TME revealed Siglec‐7 expression on CD3^+^ T cells, CD11b^+^ myeloid cells and CD56^+^ NK cells, and Siglec‐9 on CD11b^+^ cells. This result verified the broad expression of Siglec‐7 and Siglec‐9 on multiple immune cell types, thus providing potential as broad immunotherapy targets. Importantly, expression of Siglec‐9 has previously been found on tumor infiltrating T cells in non‐small cell lung cancer and melanoma.^36^, ^37^ Our results did not clearly show Siglec‐9 expression on CD3^+^ cells, but this might be because of the relatively low Siglec‐9 staining levels and higher background seen in the Siglec‐9/CD3 co‐stainings.

Interestingly, the Siglec‐7 and Siglec‐9 co‐stainings with immune cell markers showed Siglec‐7 and Siglec‐9 expression in a punctuated fashion on the membranes of multiple immune cells. Previously, our lab and others have shown that Siglec‐7 clusters on the membrane at the site of interaction with a tumor cell, which is associated with Siglec‐7 signalling.^32^, ^38^ We therefore hypothesise that Siglec‐7 and Siglec‐9 might be signalling to induce immune cell inhibition in breast cancer. Releasing the brake on effector cells such as NK cells and cytotoxic T cells by blocking Siglec‐7 could potentially increase tumor control, as it has previously been shown that Siglec‐7 can inhibit NK cell cytotoxicity and effector T cell functions.^29^, ^36^, ^37^, ^39^ However, the activation status of the Siglec‐7/9 expressing immune cells warrants further investigation, for instance, the polarisation of the CD11b^+^ cells to pro‐ or anti‐tumorigenic cells. Previous studies have shown that the presence of M2 tumor‐associated macrophages (TAMs) in breast cancer is associated with poor outcome.^40^, ^41^, ^42^, ^43^, ^44^ Moreover, a study by Hussein and Hassan (2006) clearly showed an increase in CD68^+^ myeloid cells in both the parenchyma and the stroma when going from normal breast tissue, to ductal carcinoma *in situ*, to invasive carcinoma.^45^ Interestingly, it has previously been shown that pancreatic cancer cells can drive monocyte to TAM differentiation in a Siglec‐7/9‐sialic acid‐mediated mechanism, hereby inducing immunosuppressive properties.^46^ Besides TAMs, other cells that skew the TME towards a pro‐tumorigenic environment are myeloid‐derived suppressor cells (MDSCs), which have previously been shown to express Siglecs in the glioma TME.^47^ The influence of Siglec‐7 and Siglec‐9 signalling on TAM polarisation and MDSC function in breast cancer requires more extensive investigation. Immunotherapy blocking Siglec signalling could in the future be investigated to inhibit activation of such inhibitory immune cells.

We specifically stained the breast tumor sections for Siglec‐7 and Siglec‐9 ligands using recombinant Siglec proteins, instead of focusing only at general sialoglycan expression. Siglec‐7 and Siglec‐9 ligands were found to be expressed in both TN and ER+ tumor sections. It is unknown, however, whether the simple presence of Siglec‐7 and Siglec‐9 ligands equals Siglec signalling, or whether a certain Siglec ligand threshold/density is required to induce effective Siglec signalling. Although especially tumor cells were found to express Siglec‐7 ligands and Siglec‐9 ligands, stromal cells also appeared to display Siglec‐9 ligands and to a lower extent Siglec‐7 ligands in both ER+ and TN tumors. Previous research showed ligand expression in TN breast cancer in mainly the stromal regions instead of the tumor cells, which was associated with high fibrosis.^31^ Moreover, a recent study has shown that colorectal tumor cells can increase Siglec‐7/9/E ligand expression on stromal cells to induce T cell suppression.^48^, ^49^ Induction of Siglec signalling by either EpCAM^+^ tumor cells or stromal regions remains an interesting subject for future studies.

Comparing ER+ and TN breast tumor cell lines, our data now show that overall Siglec‐7 ligand and Siglec‐9 ligand expression is higher on TN cell lines relative to ER+ cell lines. Moreover, we observed that Siglec‐7 ligand expression levels and the ability to induce Siglec‐7 signalling appear to correlate, and explains the strong Siglec‐7 signalling induction by TN cell lines. Furthermore, a threshold of Siglec‐7 ligand expression appears to be required to induce significant Siglec‐7 signalling.^32^ In line with its high Siglec‐7 ligand expression, T47D was the only ER+ cell line to pass this threshold resulting in chSiglec‐7 signalling. Using novel chSiglec‐9 Jurkat/MA reporter cells, no Siglec‐9 signalling could be detected in any of the breast cancer cell lines, even though some cell lines displayed significant levels of Siglec‐9 ligands. Possibly, differences exist in the thresholds for Siglec‐7 and Siglec‐9 signalling and/or Siglec ligand expression, density or distribution.^22^, ^32^ Alternatively, Siglec‐9 *cis*‐ligands on the chSiglec‐9 Jurkat/MA could be affecting the induction of Siglec‐9 signalling.^22^

Altogether, this study showed that Siglec‐7 and Siglec‐9 are highly expressed in TN tumors and to a lower extent in ER+ tumors on a range of immune cells. Moreover, signalling experiments showed Siglec‐7 signalling induced by breast cancer cell lines. We therefore envision that immunotherapy targeting Siglec‐7 and/or Siglec‐9 is particularly promising for TN breast cancer patients who do not respond to current treatment options with tumors presenting with high immune cell infiltration.

## Methods

### Human breast tumor sections

Coded (anonymised) primary breast tumor samples from human breast cancer patients left‐over after surgery, immediately flash‐frozen in liquid nitrogen and kept at −80°C, were used. The requirement for consent was waived by the IRB (Institutional Review Board) Radboud University Medical Center (Approval #2013/576). ER, progesterone receptor and HER2 status were established as part of the standard pathological workup and assigned to the tumors before anonymisation. None of the patients received neo‐adjuvant treatment.

### *SIGLEC7* and *SIGLEC9* expression in breast cancer samples

The UCSC Xena platform was used to retrieve *SIGLEC7* and *SIGLEC9* gene expression data in breast cancer samples.^50^ First, out of 1247 samples that were present in the TCGA Breast Cancer (BRCA) study, the primary tumor samples were selected using the ‘sample_type’ variable.

To investigate gene expression in the ER+ subtype, the ‘ER_status_nature2012’ variable was used to select samples that were ‘positive’ or ‘blank’ and the ‘breast_carinoma_oestrogen_receptor_status’ was selected to be ‘positive’.

To assess gene expression in the TN subtype, samples were selected that were ‘negative’ or ‘blank’ for the ‘ER_Status_nature2012’ variable, ‘negative’ for the ‘breast_carcinoma_oestrogen_receptor_status’ variable, ‘negative’ or ‘blank’ for ‘PR_Status_nature2012’ and ‘negative’ for ‘breast_carcinoma_progesterone_receptor_status’. Next, HER2 negative samples were chosen using ‘negative’ or ‘blank’ for ‘HER2_Final_Status_nature2012’, ‘negative’ or ‘blank’ for ‘lab_proc_her2_neu_immunohistochemistry_receptor_status’ and ‘negative’ or ‘blank’ for ‘lab_procedure_her2_neu_in_situ_hybrid_outcome_type’. Lastly, the four samples that were blank for all 3 HER2 categories were removed.

### Immunohistochemistry

For all stainings, frozen tumor tissue was cut into 5‐μm sections using a Leica CM1950 cryostat. After air‐drying the sections, different staining protocols were followed for the various proteins.

To stain for EpCAM, CD45, Siglec‐7, Siglec‐9, CD56, CD66b or CD3, the sections were blocked with Carbo‐Free Blocking Solution (SP‐5040‐125, Vector Laboratories, Newark, USA) for 30 min at room temperature (RT), washed with PBS, fixed with 1% formaldehyde for 10 min at RT and subsequently washed with PBS. Next, the sections were incubated overnight at 4°C with 1:50 mouse anti‐CD326 EpCAM Biotin (130‐113‐824, RRID:AB_2726340, Miltenyi Biotec Leiden, The Netherlands), 5 μg mL^−1^ mouse anti‐CD45 Alexa 488 (304017, RRID:AB_389314, BioLegend, San Diego, USA), 5 μg mL^−1^ mouse anti‐Siglec‐7 AF594 (NBP2‐37732AF594, Novus Biologicals, Abingdon, UK), 5 μg mL^−1^ mouse anti‐Siglec‐9 (MAB1139, RRID:AB_2270258, R&D Systems, Minneapolis, USA), 0.2 μg mL^−1^ rabbit anti‐CD56 (156r‐95, RRID:AB_2864402, Cell Marque, Rocklin, USA), 1:500 mouse anti‐CD66b FITC (IM0531U, RRID:AB_10638220, Beckman Coulter Life Sciences, Brea, USA), 10 μg mL^−1^ mouse IgG1 isotype control (400102, RRID:AB_2891079, BioLegend, San Diego, USA), or 5 μg mL^−1^ mouse IgG2a isotype control (400202, RRID:AB_2927399, BioLegend, San Diego, USA) diluted in primary antibody diluent (926001, PAD, Biolegend, San Diego, USA). To stain for CD3, sections were incubated with 1:200 rabbit anti‐CD3 (ab16669, RRID:AB_443425, AbCam, Cambridge, UK) or 5 μg mL^−1^ rabbit IgG isotype control (3900S, Cell Signalling Technology, Danvers, USA) diluted in PAD for 45 min at 37°C. The sections were then washed with PBS and endogenous peroxidase activity was quenched using 0.3% H_2_O_2_ for 15 min at RT. After washing the sections with PBS, they were incubated with BrightVision goat anti‐mouse or goat anti‐mouse/rabbit IgG HRP (DPVO110HRP, ImmunoLogic, Arnhem, The Netherlands) for 1 h at RT. The sections were washed with PBS and subsequently incubated with DAB (3,3′‐diaminobenzidine tetrahydrochloride). Enhanced Liquid Substrate System (D3939, Sigma‐Aldrich, Burlington, USA) at RT. After washing with PBS and demi water, a counterstaining with haematoxylin (S330130‐2, Agilent Dako, Santa Clara, USA) was performed. Finally, sections were dehydrated and mounted with NeoMount (1.09016.0500, Merck, Darmstadt, Germany).

To stain for CD11b, the sections were fixed with acetone for 10 min at 4°C, air‐dried and rehydrated in PBS. Next, they were blocked with Carbo‐Free Blocking Solution for 30 min at RT and incubated with 10 μg mL^−1^ mouse anti‐CD11b (GTX20183, GeneTex, Irvine, USA) in PAD overnight at 4°C. Subsequently, the same procedure was followed as described above.

To stain the tumor sections for Siglec‐7 ligands or Siglec‐9 ligands, 50 nM biotinylated human Siglec‐7 protein (SG7‐H82E7, Acro Biosystems, Newark, USA) or biotinylated human Siglec‐9 protein (SI9‐H82E9, Acro Biosystems, Newark, USA) was pre‐complexed with 50 nM Streptavidin‐HRP (21126, ThermoFisher Scientific, Waltham, USA) in Carbo‐Free Blocking Solution for 1 h at 4°C. Sections were fixed with 4% formaldehyde for 10 min at RT, washed with PBS and permeabilised with 0.1% Triton‐X in PBS for 5 min at RT. After washing the sections, they were incubated with 0.3% H_2_O_2_ for 15 min at RT, washed with PBS and blocked with Carbo‐Free Blocking Solution for 1 h at RT. Next, endogenous biotin was blocked using the Avidin/Biotin Blocking System (927301, Biolegend, San Diego, USA) according to the manufacturer's instructions. The sections were subsequently incubated with the pre‐complexed biotinylated Siglec‐7 and Siglec‐9 proteins for 1 h at RT. Next, they were washed with PBS and stained with DAB Enhanced Liquid Substrate System and processed accordingly as described above.

To stain for presence of acidic glycoproteins, frozen sections were fixed with 4% formaldehyde for 10 min at RT, washed with PBS and an alcian blue staining was done according to the manufacturer's protocol of the Alcian Blue (pH 2.5) Stain Kit (H‐3501, Vector Laboratories, Newark, USA). Sections were mounted with NeoMount.

To stain for lectin binding, frozen sections were fixed with acetone for 10 min at 4°C, air‐dried and then rehydrated with PBS. Next, the sections were blocked using 3% bovine serum albumin (BSA) for 1 h at RT. After blocking the sections, they were incubated with 5 μg mL^−1^ MALII (B‐1265‐1, Vector Laboratories, Newark, USA) or 2 μg mL^−1^ SNAI (B‐1305‐2, Vector Laboratories, Newark, USA) in 1% BSA in PBS, 1 mM CaCl_2_ and 1 mM MgCl_2_ overnight at 4°C. Subsequently, they were washed 4 times in 5 min with 1% BSA in PBS and then incubated with 1 μg mL^−1^ Streptavidin‐AF488 (S32354, ThermoFisher Scientific, Waltham, USA) in 1% BSA in PBS for 60 min at 37°C. Next, the sections were washed 4 times with 1% BSA in PBS and then stained with 0.33 μg mL^−1^ Hoechst in PBS for 5 min at RT. Sections were mounted using Fluoromount W (21634.01, Serva Electrophoresis Gmbh, Heidelberg, Germany).

Images were acquired using the Zeiss Axioimager D2 with 10× and 40× objectives with the Axiocam Icc camera (Zeiss, Oberkochen, Germany) and ZEN Pro software.

### Scoring Siglec‐7 and Siglec‐9 positivity in breast tumor sections

Siglec‐7 and Siglec‐9 expression levels in 9 TN and 8 ER+ breast tumors were scored by two researchers. Symbols (−, +, ++ and +++) are associated with extent of Siglec‐7 and Siglec‐9 expression in the tumors. ‘−’ (negative) means that the tumor is negative for the stained protein. ‘+’ (slightly positive) indicates that some positive cells can be observed within the tumor, but the vast majority is clearly negative for the stained protein. ‘++’ (positive) points out that patches within the tumor are presenting positive staining for the stained protein, but this does not represent the majority of the tumor section. ‘+++’ (highly positive) specifies that the majority of the tumor areas is positive for the stained protein.

### Immunofluorescent double staining for Siglec‐7 or Siglec‐9 with CD3, CD11b or CD56

To perform double stainings for Siglec‐7 or Siglec‐9 with either CD3 or CD56, tumor sections were fixed with 1% formaldehyde for 10 min at RT, washed with PBS and blocked with Carbo‐Free Blocking Solution for 30 min at RT. After washing with PBS, sections were incubated with 5 μg mL^−1^ mouse anti‐Siglec‐7 (MAB1138, RRID:AB_2286070, R&D Systems, Minneapolis, USA), 5 μg mL^−1^ mouse anti‐Siglec‐9 (MAB1139, RRID:AB_2270258, R&D Systems, Minneapolis, USA), 5 μg mL^−1^ mIgG1 or 5 μg mL^−1^ mIgG2a diluted in PAD overnight at 4°C. Subsequently, the sections were washed with PBS and stained with 5 μg mL^−1^ donkey anti‐mouse‐Cy3 (715‐166‐151, RRID:AB_2340817, Jackson ImmunoResearch, Cambridgeshire, UK) for 45 min at 37°C. After washing with PBS, the sections were incubated with 1:200 rabbit anti‐CD3 (ab16669, RRID:AB_443425, Abcam, Cambridge, UK), 0.22 μg mL^−1^ rabbit anti‐CD56 (156R‐95, RRID:AB_2864402, Cell Marque, Rocklin, USA), or 5 μg mL^−1^ rabbit IgG isotype control in PAD for 45 min at 37°C. Next, the sections were washed and incubated with 5 μg mL^−1^ donkey anti‐rabbit‐AF488 (A21206, RRID:AB_2535792, ThermoFisher Scientific, Waltham, USA) for 45 min at 37°C. Sections were subsequently washed with PBS and incubated for 5 min with TrueView Reagent (SP‐8400‐15, Vector Laboratories, Newark, USA) at RT. After washing with PBS, nuclei were stained with 0.33 μg mL^−1^ Hoechst (94 403, Sigma‐Aldrich, Burlington, USA) in PBS for 5 min at RT.

To perform double stainings for Siglec‐7 or Siglec‐9 with CD11b, sections were processed and stained for Siglec‐7 and Siglec‐9 as described above. After staining with the donkey anti‐mouse‐Cy3 antibody, sections were washed and incubated with 10 μg mL^−1^ mouse anti‐CD11b‐AF647 (FAB1699R, R&D Systems, Minneapolis, USA) or 10 μg mL^−1^ mouse IgG1‐AF647 (400130, RRID:AB_2800436, Biolegend, San Diego, USA). Afterwards, sections were washed with PBS, and stainings were proceeded with the TrueView Reagent as described above.

Sections were mounted using Vectashield Vibrance Antiface mounting medium (H‐1700, Vector Laboratories, Newark, USA). Images were acquired with the same system as described above, using a 63× objective.

### Cell culture

HCC1143 (CRL‐2321, RRID:CVCL_1245, LGC Standards, Teddington, UK), HCC1937 (CRL‐2336, RRID:CVCL_0290, LGC Standards, Teddington, UK), BT‐474 (HTB‐20, RRID:CVCL_0179, LGC Standards, Teddington, UK) and T47D (HTB‐133, CVCL_0553, LGC Standards, Teddington, UK) cell lines were cultured in RPMI Medium 1640 (42401042, ThermoFisher Scientific, Waltham, USA) with 10% FCS (F7524, Sigma‐Aldrich, Burlington, USA), 1% PenStrep (15140163, ThermoFisher Scientific, Waltham, USA), 1% MEM nonessential amino acids (11140035, ThermoFisher Scientific, Waltham, USA) and 2 mmol L^−1^ L‐glutamine (BEBP17‐605E, Lonza, Basel, Switzerland). MCF7 cells (HTB‐22, RRID:CVCL_0031, LGC Standards, Teddington, UK) were cultured in Dulbecco's Modifed Eagle Medium (DMEM) with GlutaMAX (32430100, ThermoFisher Scientific, Waltham, USA) supplemented with 10% FCS, 1% PenStrep and 6 ng mL^−1^ insulin (19278, Sigma‐Aldrich, Burlington, USA). MDA‐MB‐175 (HTB‐26, RRID:CVCL_1400, ATCC, Manassas, USA) and MDA‐MB‐468 (HTB‐132, RRID:CVCL_0419, ATCC, Manassas, USA) cells were grown in DMEM GlutaMAX with 10% FCS, 1% PenStrep, 1 mmol L^−1^ Sodium Pyruvate (11360039, ThermoFisher Scientific, Waltham, USA) and 1% MEM nonessential amino acids. MDA‐MB‐231 (HTB‐26, RRID:CVCL_0062, LGC Standards, Teddington, UK) cells were cultured in DMEM GlutaMAX supplemented with 10% FCS, 1% PenStrep and 1 mmol L^−1^ Sodium Pyruvate. chSiglec‐9 Jurkat/MA cells were generated similarly as previously described for chSiglec‐7 Jurkat/MA cells.^32^ chSiglec‐7 Jurkat/MA cells and chSiglec‐9 Jurkat/MA cells were cultured in Iscove's Modifed Dulbecco's Medium (21980065, IMDM, ThermoFisher Scientific, Waltham, USA) supplemented with 8% FCS, 500 μg mL^−1^ Hygromycin B (10687010, ThermoFisher Scientific, Waltham, USA) and 1 mg L^−1^ Geneticin (10131027, ThermoFisher Scientific, Waltham, USA).^50^

All cell lines were grown under 5% CO_2_ and 37°C incubation conditions. HCC1143, HCC1937, BT‐474 and MDA‐MB‐175 were kindly provided by J Martens (ErasmusMC, Rotterdam, The Netherlands).

### Flow cytometric analysis of Siglec ligands

To determine Siglec‐7 and Siglec‐9 ligand expression on cell lines, a recombinant Siglec‐7 Fc, Siglec‐9 Fc or IgG1 Fc staining was performed as previously described.^32^ Briefly, cells were stained with a viability dye, recombinant Siglec Fc proteins (Siglec‐7 Fc: 1138‐SL‐050, Siglec‐9 Fc: 1139‐SL‐050, and IgG1 Fc: 110‐HG‐100, R&D Systems, Minneapolis, USA) were pre‐complexed with Alexa Fluor 488‐conjugated goat anti‐human IgG (H + L) (A‐11013, ThermoFisher Scientific, Waltham, USA) in Hank's buffered salt solution (14‐025‐092, ThermoFisher Scientific, Waltham, USA) and subsequently used to stain the cells. Cells were acquired on a BD FACS CantoII Flow Cytometer with the BD FACSdiva software. Data were analysed using the FlowJo software (RRID:SCR_008520, version 10.7.0, Tree Star Inc.). Cells were pretreated for 3 days with 31.25 μM SiaFEt or DMSO as vehicle control.

### Luciferase assays

To determine the ability of breast cancer tumor cell lines to induce Siglec signalling, Jurkat/MA cells were harvested at a density between 400 000 and 800 000 cells mL^−1^. Next, chimeric Siglec‐7 or chimeric Siglec‐9 Jurkat/MA reporter cells and tumor cells were washed with 0.5% FBS‐containing phenol red‐free IMDM.^32^

To investigate chSiglec‐7 signalling, assays were performed in cell culture‐treated flat‐bottom microplates (10695951, Corning Costar, Glendale, USA). As controls, the chSiglec‐7 Jurkat/MA cells were cultured with 5 μg mL^−1^ Neu5Ac‐polyacrylamide‐biotin (0035‐BP, GlycoNZ, Auckland, New Zealand) or stimulated with 5 ng mL^−1^ Phorbol 12‐myristate 13‐acetate (PMA, tlrl‐pma Invivogen, Toulouse, France) together with 0.5 μM ionomycin (I0634‐1MG, Sigma‐Aldrich, Burlington, USA) for 16 h. To induce anti‐Siglec‐7 mediated Siglec‐7 signalling, wells were coated with 5 μg mL^−1^ mouse anti‐Siglec‐7 (Custom made Ultra‐LEAF purified clone S7.7, Biolegend, San Diego, USA), or 5 μg mL^−1^ mouse IgG1 isotype control (clone MOPC‐21) for 2 h at 37°C. Next, wells were washed with PBS, and cells were plated and incubated for 16 h.

To assess chSiglec‐9 signalling, assays were performed in high‐binding flat‐bottom microplates (655092, Greinier Bio‐One, Kremsmünster, Austria). As controls, wells were coated with 5 μg mL^−1^ mouse anti‐Siglec‐9 (MAB1139, clone 191240, R&D Systems, Minneapolis, USA), with 5 μg mL^−1^ mouse IgG2a isotype control (400202, clone MOPC‐173, Biolegend, San Diego, USA) or with 5 μg mL^−1^ SiaLex‐BP (0062‐BP, GlycoNZ, Auckland, New Zealand) for 2 h at 37°C. After washing with PBS, wells were blocked with Carbo‐Free Blocking Solution for 1 h at RT. Next, wells were washed with PBS, and cells were plated and incubated for 16 h.

Co‐cultures were set up containing 1:1 chSiglec Jurkat/MA cells with tumor cells and cultured for 16 h in 0.5% FBS‐containing phenol red‐free IMDM. Tumor cells were pretreated for 3 days with 31.5 μM SiaFEt or DMSO as vehicle control.

Finally, luminescence was measured using the Bright‐Glo™ Luciferase Assay System (E2620, Promega, Madison, USA) according to the manufacturer's protocol with a Victor3 multilabel plate reader (PerkinElmer). Luminescence background values of medium only controls were subtracted from all measurements.

### Statistics

Statistical significance was assessed using Prism 9.0.0 (GraphPad Software, RRID:SCR_002798). A Mann–Whitney *U*‐test was performed to assess statistically significant differences between *SIGLEC7* and *SIGLEC9* RNA expression. To assess differences in Siglec‐7 signalling, matched one‐way ANOVAs were performed followed by Tukey's multiple comparison test, on log‐transformed raw data to ensure normality of distribution. A *P*‐value threshold of < 0.05 was considered to be statistically significant (*P* < 0.05*, *P* < 0.01**, *P* < 0.001***, *P* < 0.0001****).

## Author contributions

**Eline JH van Houtum:** Conceptualization; data curation; formal analysis; funding acquisition; investigation; methodology; project administration; visualization; writing – original draft; writing – review and editing. **Anne HC Valk:** Formal analysis; investigation; methodology; project administration; visualization; writing – original draft; writing – review and editing. **Daniel Granado:** Conceptualization; formal analysis; funding acquisition; investigation; methodology; project administration; writing – review and editing. **Jasper Lok:** Data curation; formal analysis; investigation; methodology; validation; visualization; writing – review and editing. **Lune van den Bogaard:** Formal analysis; investigation; methodology; validation; writing – review and editing. **Naomi Remkes:** Conceptualization; formal analysis; investigation; methodology; project administration; validation; writing – review and editing. **Jesper van Eck van der Sluijs:** Conceptualization; formal analysis; investigation; methodology; project administration; supervision; writing – review and editing. **Paul N Span:** Conceptualization; formal analysis; investigation; methodology; resources; supervision; validation; writing – review and editing. **Lenneke AM Cornelissen:** Conceptualization; funding acquisition; methodology; supervision; visualization; writing – original draft; writing – review and editing. **Gosse J Adema:** Conceptualization; funding acquisition; investigation; methodology; project administration; resources; supervision; validation; writing – original draft; writing – review and editing.

## Conflict of interest

The authors declare no conflict of interest.

## Supporting information


Supplementary figure 1
Supplementary figure 2
Supplementary figure 3
Supplementary figure 4
Supplementary figure 5
Supplementary figure 6
Supplementary figure 7

## Data Availability

Data generated during this study are available upon reasonable request.
