# Supplementary material for: Siglec‐7 and Siglec‐9 expression in primary triple negative and oestrogen receptor positive breast cancer and in vitro signalling
Source: Clin Transl Immunology. 2024 Sep 6;13(9):e1524. doi: 10.1002/cti2.1524 (PMC11378723; doi:10.1002/cti2.1524)
Supplement: Supplementary file 1 — Supplementary figure 1 Supplementary figure 2 Supplementary figure 3 Supplementary figure 4 Supplementary figure 5 Supplementary figure 6 Supplementary figure 7 [file CTI2-13-e1524-s001.pdf]

Supporting information

Siglec-7 and Siglec-9 expression in primary triple negative and oestrogen receptor positive breast cancer and *in vitro* signalling

Eline JH van Houtum[1], Anne HC Valk[1], Daniel Granado[1], Jasper Lok[1], Lune van den Bogaard[1], Naomi Remkes[1], Jesper van Eck van der Sluijs[1], Paul N Span[1], Lenneke AM Cornelissen[1], Gosse J Adema[1]

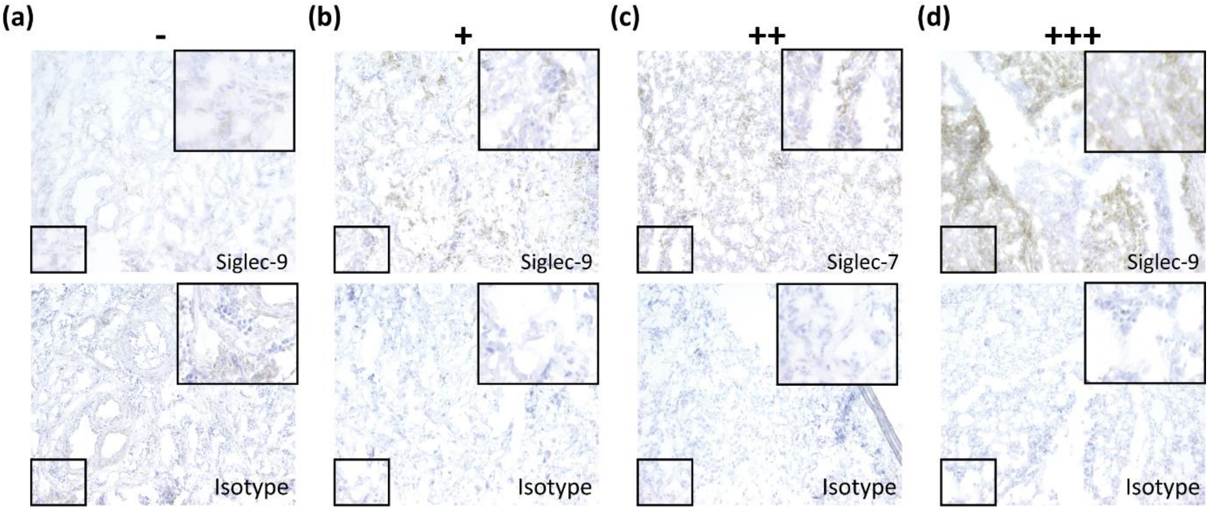

Supplementary figure 1. Representative images illustrating scoring of Siglec-7 and Siglec-9 stainings. Level of Siglec-7 and Siglec-9 expression in TN and ER+ breast tumors was scored by two researchers. **(a)** Representative image of '-', indicating negative staining (tumor #13), **(b)** representative image of '+', indicating a slightly positive staining (tumor 7), **(c)** representative image of '++', indicating a positive staining (tumor 6) and **(d)** representative image of '+++', indicating a highly positive staining (tumor 1).

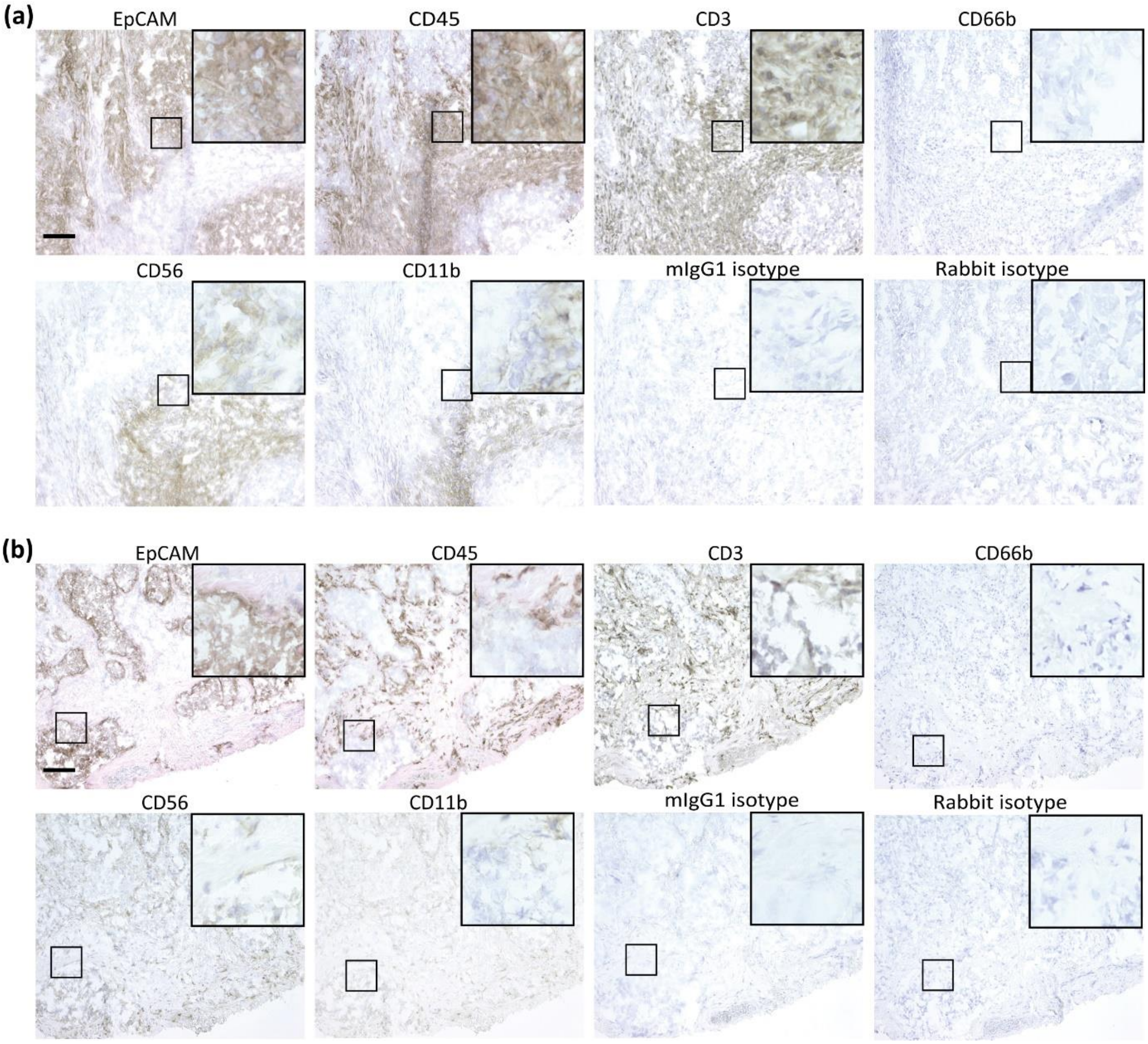

Supplementary figure 2. TN and ER+ tumors are infiltrated with CD3^{+}, CD56^{+} and CD11b^{+} cells **(a)** TN tumors and **(b)** ER+ tumors were stained for EpCAM, CD45, CD3, CD66b, CD56, CD11b, mIgG1 (isotype control for EpCAM, CD45, CD66b, CD11b) and rabbit isotype (isotype control for CD3 and CD56). For each subtype, one representative tumor is shown. The scale bars indicate 100 µm.

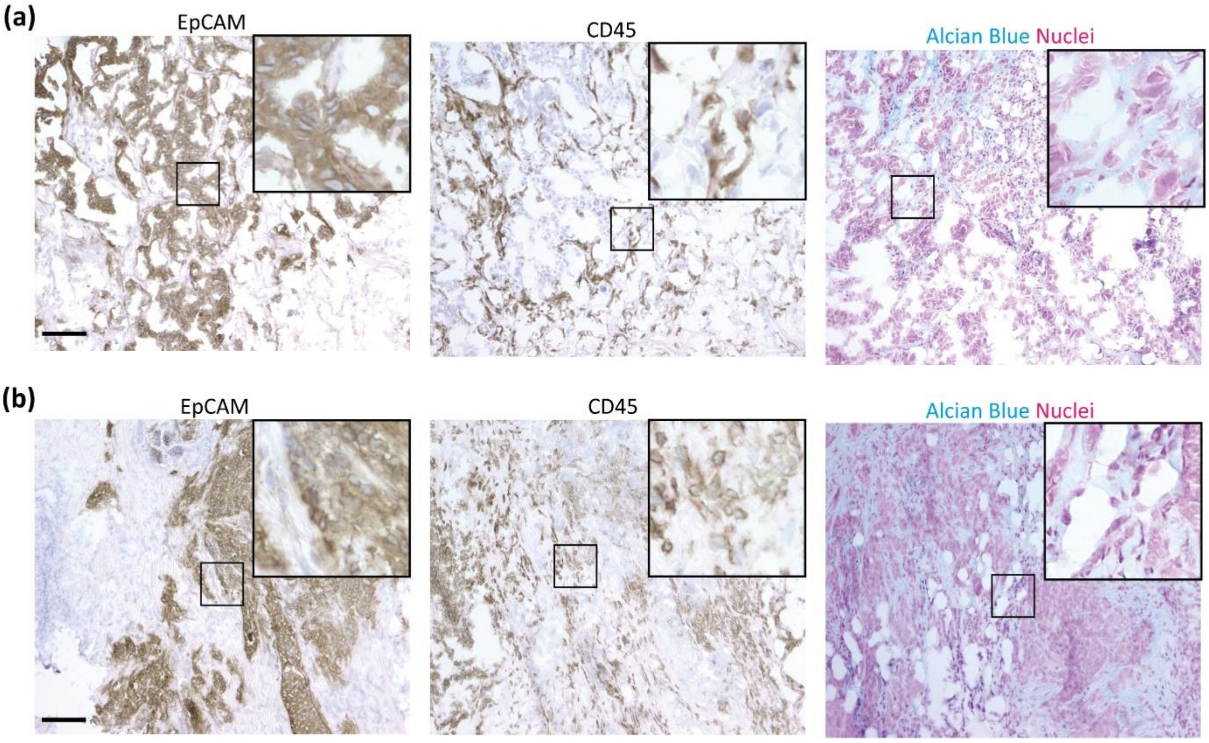

Supplementary figure 3. TN and ER+ tumors express sialoglycans. Stainings were performed for EpCAM, CD45, and acidic glycoproteins (Alcian blue staining), and a representative **(a)** TN tumor and a representative **(b)** ER+ tumor are shown. The scale bar shows 100 µm.

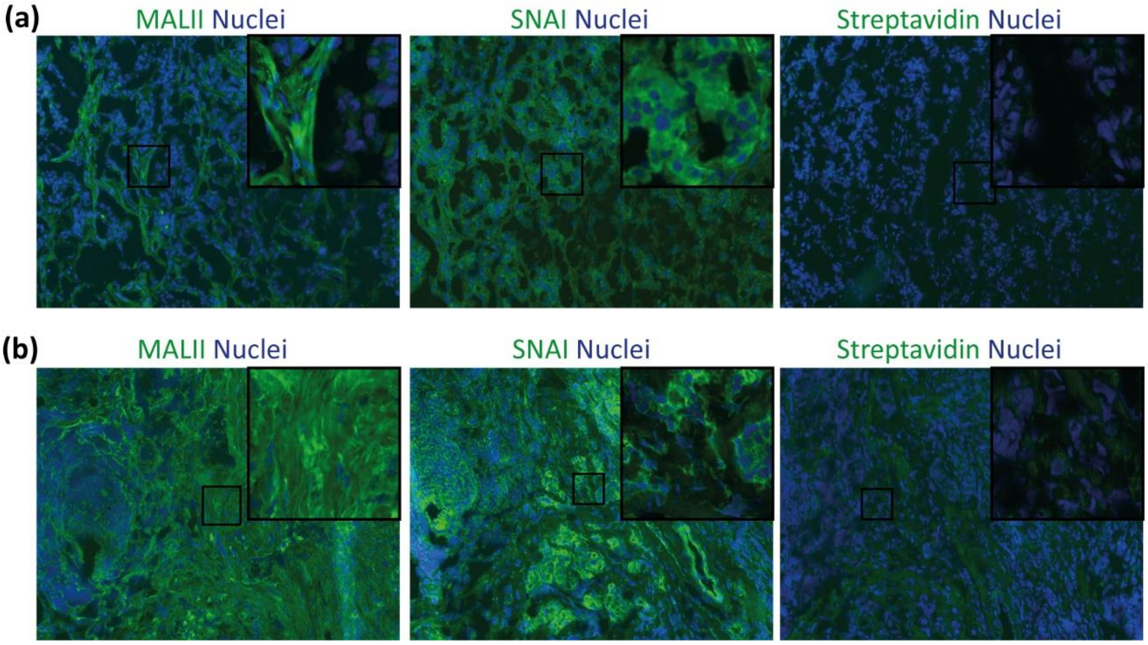

Supplementary figure 4. TN and ER+ tumors express α-2,3 and α-2,6 linked sialic acid. A TN tumor and a ER+ tumor were stained for α-2,3 linked sialic acid with MALII lectin and α-2,6 linked sialic acid with SNAI lectin. A representative **(a)** TN and **(b)** ER+ tumor are shown.

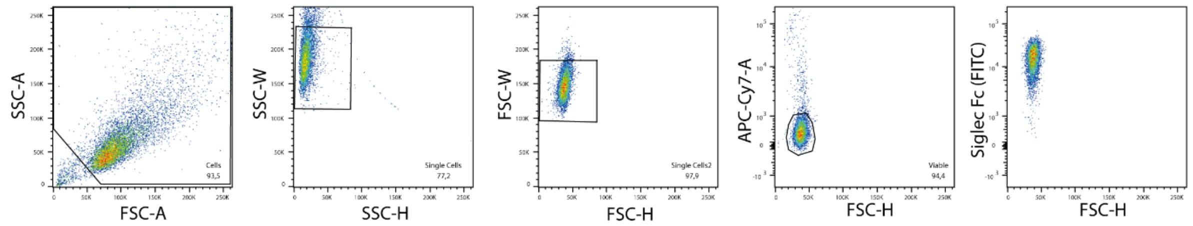

Supplementary figure 5. Gating strategy for detection of Siglec-7/9 ligand expression by breast cancer cell lines. First, debris was excluded (SSC-A vs FSC-A), then single cells were gated (first SSC-W vs SSC-H and then FSC-W vs FSC-H), and finally viable cells were gated (APC-Cy7-A vs FSC-H). A representative gating strategy for HCC1937 cells is shown. All other cell lines are gated similarly.

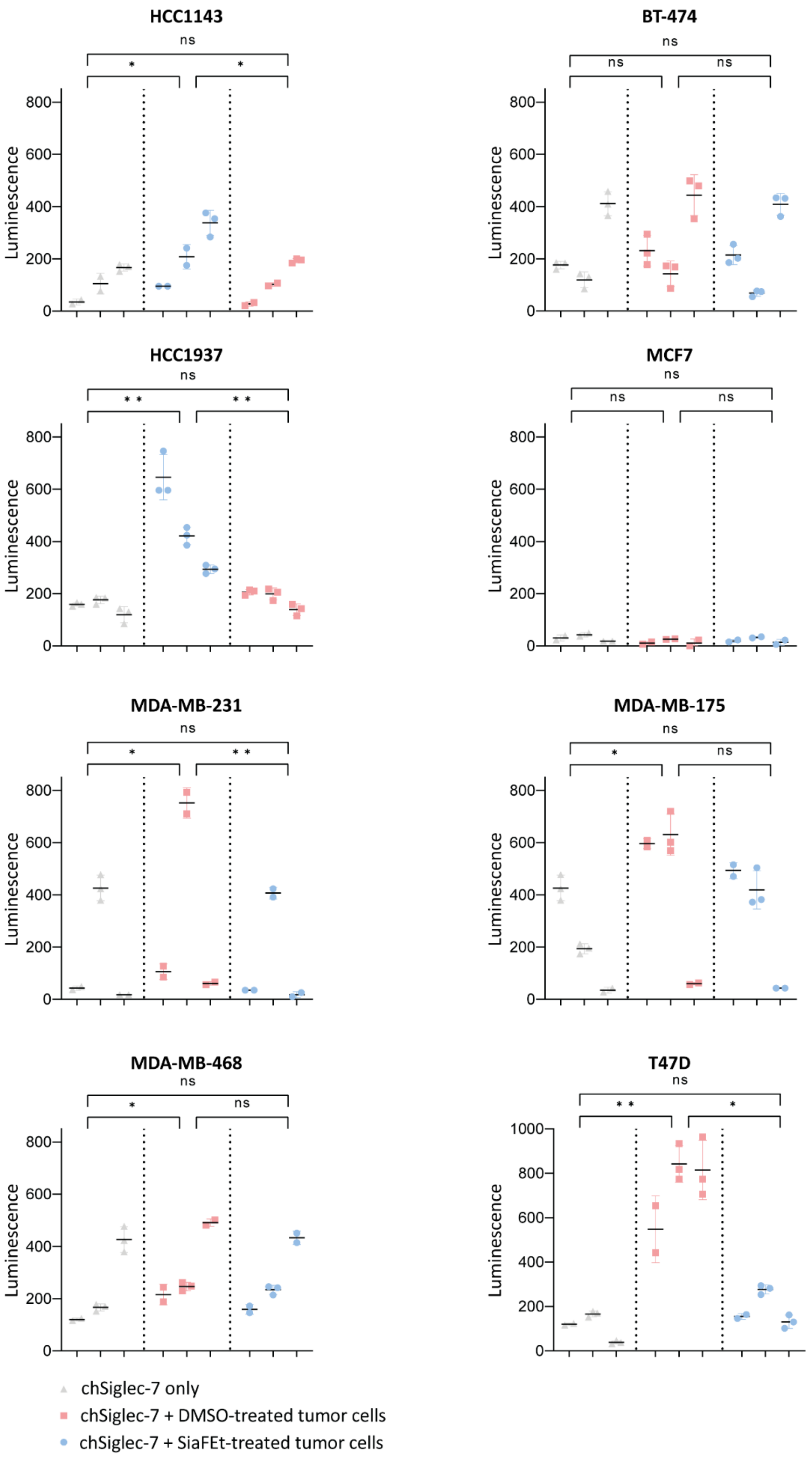

Supplementary figure 6. chSiglec-7 signaling is induced by three TN and one ER+ tumor cell line. luciferase assays were performed after 16h of culture of chSiglec-7 cells only, or of chSiglec-7 cells co-cultured with SiaFEt- or DMSO-treated tumor cells (n = 3, each containing 2-3 technical replicates). Matched one-way ANOVAs were performed on log-transformed raw data. (Adjusted P<0.05 *, $P < 0.01$ **).

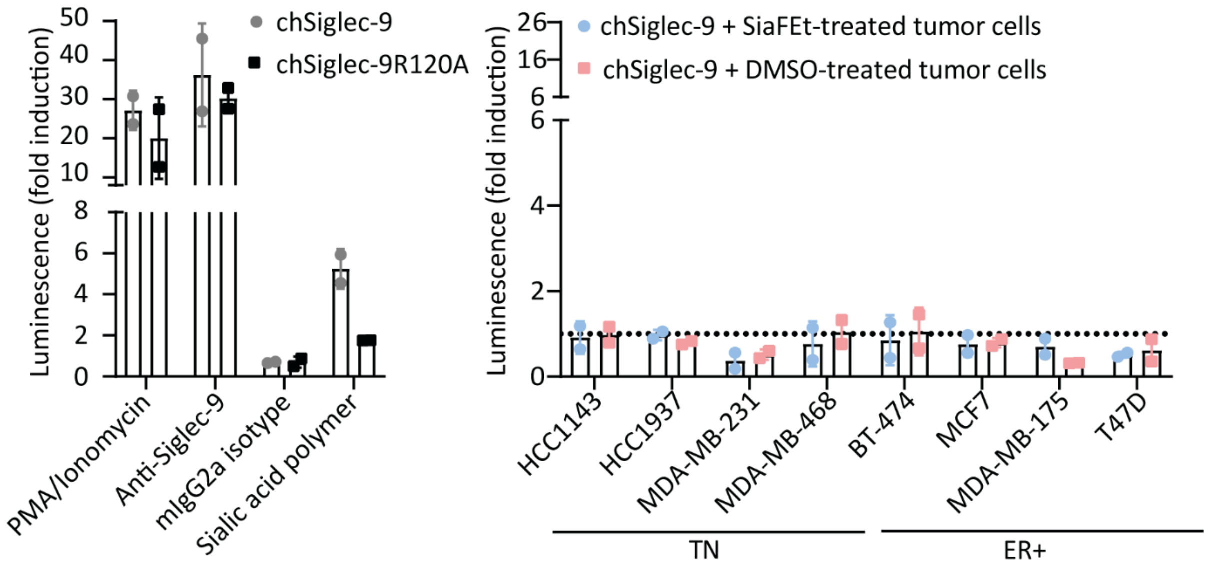

Supplementary figure 7. Breast cancer cell lines do not induce Siglec-9 signaling. chSiglec-9 cells were validated for functionality by culture with PMA/ionomycin, coated anti-Siglec-9 antibody or respective mIgG2a isotype control, or with sialic acid polymer (left panel, showing two technical replicates of a representative assay). chSiglec-9 Jurkat/MA cells were co-cultured with SiaFEt- or DMSO-treated tumor cell lines and subsequently chSiglec-9 signaling was assessed by luciferase assays (right panel). Luciferase assays were repeated 2 times, each containing 1-2 technical replicates per condition, bars represent mean ± SD. Luminescence values are normalized to those of unstimulated chSiglec-9 cells.
